# *In vitro* assessment of *Bacillus subtilis* FJ3 affirms its biocontrol and plant growth promoting potential

**DOI:** 10.3389/fpls.2023.1205894

**Published:** 2023-07-18

**Authors:** Faisal Jan, Hamza Arshad, Mehreen Ahad, Asif Jamal, Donald L. Smith

**Affiliations:** ^1^ Department of Microbiology, Quaid-i-Azam University, Islamabad, Pakistan; ^2^ Department of Plant Science, McGill University, Ste. Anne de Bellevue, QC, Canada

**Keywords:** *Bacillus subtilis* FJ3, lipopeptides, antifungal, plant growth promoting traits and agriculture, biocontrol

## Abstract

*Bacillus* species and their metabolites have potential alternative uses as chemical pesticides that can limit the growth of potential plant pathogens and enhance crop productivity. The aim of this study was to investigate the potential of *Bacillus subtilis* FJ3 for promoting plant growth and controlling fungal plant pathogens. The study evaluated the ability of the strain to promote plant growth *in vitro* by characterizing its growth-promoting traits, which included the production of hydrolytic enzymes, indole acetic acid, siderophores, biofilm formation, and phosphate solubilization. Polymerase Chain Reaction (PCR) testing revealed that strain FJ3 has the potential to produce lipopeptides such as fengycin, surfactin, mycosubtilin, and pilpastatin. Through *in vitro* antagonism testing it was demonstrated that strain FJ3 is able to inhibit *Fusarium oxysporum* by 52% compared to the untreated control and was antagonistic against *Aspergillus flavus*, *Aspergillus niger*, and *Rhizopus oryzae* using a dual method. The minimum inhibitory concentration of *Bacillus* crude extract resulted in a 92%, 90%, 81.5%, and 56% growth inhibition of *Fusarium oxysporum*, *A. niger*, *A. flavus*, and *Rhizopus oryzae*, respectively. In FT-IR and GC-MS analysis of crude LPs extract, the transmission and mass spectrum confirmed the existence of aforesaid lipopeptides containing β-fatty acids with chain lengths ranging from C14 to C21 in which the majority were saturated fatty acids. Greenhouse experimentation revealed that *Bacillus* strain FJ3 and its metabolites significantly diminished the disease incidence with an average reduction of 31.56%. In sterilized soil, FJ3 and its metabolites caused 24.01% and 10.46% growth promotion, respectively, in chickpea. The results demonstrated that *Bacillus* strain FJ3 has broad-spectrum antifungal and plant growth-promoting applications and could be a promising candidate for development into a commercialized biobased product for use in sustainable agriculture practice.

## 1 Introduction

With the increasing human population the need for increased food production and to ensure global food security will continue to mount. Discrepancies between food supply and demand create an imbalance that has dramatically worsened during the past few decades (Pinstrup-Andersen and Pandya-Lorch, 1998). During the next 35 years, it seems likely that food security, water scarcity, and environmental pollution will become increasingly important issues with regard to crop agriculture (Shiferaw et al., 2013). Numerous biotic and abiotic factors are responsible for productivity losses each year. Reductions in crop yield occur both qualitatively and quantitatively either in the field (pre-harvest) or during storage (post-harvest) due to pests and pathogens. Plant pathogens are major threats to agricultural outputs that range in complexity from the simplest viroid to more sophisticated pathogens such as bacteria, viruses, fungi, nematodes, and oomycetes that cause a wide range of plant infections (Seyi-Amole and Onilude, 2021). Recent research indicated that phytopathogenic fungi are responsible for 40% of crop loss worldwide at pre- and post-harvesting stages (De Angelis et al., 2022). Fungal pathogens are very problematic because of their adaptability patterns and capacity to persist in low-nutrient environments (Paulussen et al., 2017). Being an agricultural economy, Pakistan faces huge losses due to plant infections, particularly by *Fusarium* and *Aspergillus* species. *Fusarium* spp. causes infections such as root rot, crown rot, and vascular wilts in more than 2000 plant species (Zehra et al., 2022). It causes postharvest loss of grains, pulses, dry fruits, and spices by secreting several types of mycotoxins (patulin, aflatoxins, ochratoxins, zearalenone, and fumonisin deoxynivalenol), that are not only food poisoning agents but can also cause cancer, kidney failure, liver damage, and paralysis in humans (Awuchi et al., 2021).

Existing agriculture management strategies chiefly rely on synthetic chemicals that are either recalcitrant or may create a significant ecological disturbance. Under these circumstances, promotion of green technologies and bio-based products need to be promoted (Gontard et al., 2018). Furthermore, excessive input of pesticides and antimicrobials affects the density and diversity of beneficial microbial flora. Therefore, there is an urgent need to shift from conventional agricultural methods to “Green” and sustainable processes/products (Singh et al., 2019).

*Bacillus*-based biocontrol products are considered essential as biopesticides against a wide variety of plant pathogens. Typically, *Bacillus* is given priority over other biocontrol agents due to some of its basic properties such as rapid replication, resistance to adverse environmental conditions, and development and longevity of spores (Albayrak, 2019). *Bacillus* species isolated from rhizospheric soil can act as an effective plant growth promoter, induce systemic resistance in the host plant, and produce a broad range of antimicrobial compounds such as antibiotics, lipopeptides, and enzymes (Miljaković et al., 2020). Moreover, they act as major competitors for space and nutrients with pathogens in the soil (Shafi et al., 2017). Currently, a number of registered products and those in the development stage are derived from *Bacillus* species. Reported biocontrol agents (BCAs) from *Bacillus* species such as *Bacillus subtilis*, *Bacillus pumilis*, and *Bacillus thuriengiensis* are characterized by their antagonist activity and manifest fungistatic and fungicidal effects (Rajkumari and Pandey, 2019). *Bacillus* spp. support plant growth through phosphate solubilization, nitrogen fixation, and phytohormone synthesis, in addition to being the most promising biocontrol agents (Khan et al., 2022).

*Bacillus* spp. antagonist mechanism is predicted to be either by the production of cyclic lipopeptides (LPs) such as fengycin or plipastatin, surfactin, iturin, bacillomycin, mycosubtilin or through production of lytic enzymes such as chitinases, cellulase, endoglucanase, or hemicellulase which suppresses the growth of pathogens (Dimkić et al., 2022). LPs in *Bacillus* are synthesized by multi-modular enzyme systems known as non-ribosomal peptide synthetases (NRPS) by the thio-template mechanism (Caulier et al., 2019). Rearrangement of the modular organization of these enzymes within the NRPS genetic clusters is responsible for the production of a wide variety of lipopeptides. Among all the diversity, LPs of the families surfactin, iturin, and fengycin or plipastatin are regarded as the most active. These lipopeptides not only manifest antifungal activities but also play crucial roles in root colonization and plant growth-promoting activities (Nahas et al., 2021).

*Bacillus* subtilis strains are potentially effective and environmentally friendly agents for disease control. Recent studies have shown that treating these strains with nanoparticles coated with gold, aluminum, and silver can promote plant growth and prevent the growth of harmful fungi in the rhizosphere, indicating their potential as nano-biofertilizers (do Espirito Santo Pereira et al., 2021). *Bacillus subtilis* strains can activate natural defense mechanisms in plants against a wide range of pathogens, making them useful biocontrol agents (Hashem et al., 2019). They are considered safe for use in the food industry and are easy to formulate and store because of their endospore formation ability as their endospores are resistant to heat, UV radiation, organic solvents, and desiccation. Furthermore, *B. subtilis* strains can maintain their ability to activate plant defense responses under adverse conditions (Zhang et al., 2020). Considering these issues, the current research was focused on the isolation and characterization of lipopeptide-producing *Bacillus* strains as biocontrol agents against some of the most devastating plant pathogens. The ALPs were characterized and tested *in-vitro* against selected fungal pathogens and their plant growth stimulatory role was estimated.

## 2 Materials and methods

### 2.1 Isolation of *Fusarium oxysporum* and *Bacillus* spp.

*Fusarium oxysporum* was isolated from chickpea plants, from Bakkar (Pakistan) and was used in the present study. The isolated fungus was stored on sabouraud dextrose agar (SDA) at 4°C. For further experimentation, the isolate was subcultured on an SDA medium at 28 ± 1°C for 7 days.

The 13 *Bacillus* strains scrutinized in this study were isolated from the soil samples of various agricultural fields of the Swat district. Preliminary screening was performed on a minimal salt medium, which was used for the enrichment of samples and 1 gram of soil was suspended in 10 mL of sterile distilled water and serially diluted. In total, 9 dilutions of each sample were made. An aliquot of 100 µL from 10^−3^ and 10^−9^ dilutions was distributed on nutrient agar and Hi-chrome *Bacillus* agar plates for culturing at 37°C for 24 h. Then, morphological and biochemical identification was carried out via microscopy and biochemical tests.

### 2.2 Molecular identification of fungal and bacterial isolates

The bacterial strains were selected based on preliminary screening. The genomic DNA of selected bacterial strains and isolated fungal strains were extracted through the PCI (phenol, chloroform, isoamyl) method (Munir et al., 2018). Identification of fungal strains was validated by 18S rDNA and 16S rDNA for bacteria using universal primer (Chen et al., 2014) sequences. Sequencing was done at the Alpha genomics. The sequences were edited using the MEGA software version X. The nucleotide was compared to the NCBI database through the Basic Local Alignment Search Tool (NCBI) algorithm. The sequences were further submitted to GeneBank for accession number. In order to check the taxonomic placement of test organisms a phylogenetic tree was constructed on MEGA software version X (Dissanayake et al., 2020).

### 2.3 Plant growth-promoting traits

#### 2.3.1 Phosphate solubilization test

NBRIP agar medium was utilized to evaluate the potential of a selected bacterial strain to solubilize phosphate. The medium comprised the following: 10 g L^−1^ glucose, 5 g L^−1^ MgCl_2_.6H_2_O, 0.25 g L^−1^ MgSO_4_.7H_2_O, 5 g L^−1^ Ca_3_(PO_4_)_2_, 0.2 g L^−1^ KCL, 0.1 g L^−1^ (NH_4_)_2_SO_4_, 15% agar, and pH 7. Then, 5% tri-calcium phosphate was added to the medium, and bacterial strains were spot-inoculated on the NBRIP agar medium, followed by incubation at 30°C for 7 days (Mohamed et al., 2019).

#### 2.3.2 Siderophore production test

Chrome azurol sulphonate agar medium was used to identify siderophore production by selected bacterial strains. After 5 days of incubation at 28°C, the change in medium color from green to yellow indicates positive results (Grobelak and Hiller, 2017).

#### 2.3.3 Indole acetic acid production test

Indole acetic acid production was determined by inoculating selected bacterial strains in 30 mL of LB broth supplemented with tryptophan and slowly shaken at 28°C for three to five days. After incubation, the broth was subjected to centrifugation at 10,000 rpm and 4°C for 10 minutes. A two mL portion of the cell-free supernatant was mixed with 2 mL of Salkowski reagent, which is composed of 35% HClO_4_ and 0.5 M FeCl_3_ (Ullah and Bano, 2019). The mixture was then kept in the dark at room temperature for 20 to 30 min; the appearance of a pink color indicated the production of IAA.

### 2.4 Antimicrobial traits

#### 2.4.1 Hydrolytic enzymes assays

Isolated *Bacillus* strains were assayed for the production of protease, cellulase (endoglucanase), and amylase enzymes following the method of Durairaj et al. (2017). Qualitative protease activity was performed on Modified Basal Media (MM) containing 5g L^−1^ casein. The ability of the bacterial strains to produce cellulase was evaluated on plates containing 1% carboxymethylcellulose and 1M NaCl while the starch iodine method was employed for amylase assay.

#### 2.4.2 Biofilm assay

The Congo Red assay, described by Freeman et al. (1989), was used for confirmation of biofilm formation. The selected bacterial strain was streaked onto Congo Red agar, which comprised brain heart infusion (BHI) agar, sucrose, and Congo Red dye. The plate was then incubated at 37°C for 48 h.

#### 2.4.3 Amplification of NRPS genes in *Bacillus* strain

Polymerase Chain Reaction (PCR) testing was performed to identify the lipopeptide biosynthesis genes for fengycin, plipastatin, surfactin, and mycosubtilin. Primers were checked on the NRPS-producing *Bacillus* strain FJ3. Primers were ordered according to Tapi et al. (2010) from Macrogen. A list of the primers is given in **Table 1**. Genomic DNA concentration from each extract was evaluated from Nanodrop. The final DNA dilution used for PCR was between 10 and 30 ng µL^−1^.

**Table 1:** List of primers and their characteristics ordered for detection of lipopeptide biosynthetic genes (Tapi et al., 2010).

	Primer Name	Primer Sequence 3’–5’	Length	Amplified Product Size
**Surfactin**	As1-F	CGCGGMTACCGVATYGAGC	19	419,422,425,431bp
	Ts2-R	AATBCCTTTBTWDGAATGTCCGCC	23	
**Fengycin**	Af2-F	GAATAYMTCGGMCGTMTKGA	20	443,452bp
	Tf1-R	GCTTTWADKGAATSBCCGCC	20	455bp
**Mycosubtilin**	Am1-F	CAKCARGTSAAAATYCGMGG	20	416,419bp
	Tm2-R	CCDASATCAAARAADTTATC	20	
**Plipastatin**	Ap1-F	AGMCAGCKSGCMASATCMCC	20	893,959,929bp
	Tp2-R	GCKATWWTGAARRCCGGCGG	20	

PCR conditions for each set of primers were experimentally determined by running an annealing temperature gradient in a gradient thermocycler PCR (Bio-Rad). The thermal cycler was programmed for 30 cycles of PCR, including initial denaturation at 94°C for 3 min, followed by a denaturation step at 94°C for 1 min. The annealing temperature for the fengycin gene was set at 45°C, for plipastatin at 58°C, and surfactin at 43°C while for mycosubtilin it was set at 45°C. Extension for each set of primers was done at 72°C for 45 sec, (except for plipastatin for 75 sec at 72°C), followed by a final extension step of 72°C for 1 min (Caracciolo, 2018).

Three μL of amplified PCR product mixed with loading dye was loaded into the Agarose gel 2X stained with Et.Br. For band size estimation, a gene ladder of 1 kb was used. A DNA ladder of 1 kb was ordered from Solis Bio Dyne. The gels were observed under a UV illuminator at low and high resolutions to detect DNA bands, and photographs were captured using a Kodak DC 290 digital camera from New York, USA. Positive PCR results were indicated when bands of appropriate size were visualized under a UV illuminator (Weal Tax).

### 2.5 Determination of *in vitro* antifungal activity

#### 2.5.1 Agar well diffusion method and dual culture assay

The antagonistic effect of a bacterial strain against isolated fungal strains of *Fusarium oxysporum* was assessed. The spore suspension method was employed for fungal culturing (Jangir et al., 2018). The pathogenic fungal strains were transferred to sabouraud dextrose agar (SDA) plates prior to use. The cell-free supernatants of bacterial strains were taken after centrifugation. The *Bacillus* strain (72 h growth) was inoculated at an equidistant central point via the agar well diffusion method. The plates were sealed with parafilm and incubated at 28 ± 1°C. The inhibition zones were recorded using a scale (Chopra et al., 2020). Moreover, growth inhibition of mycelium by the potentially best *Bacillus* sp. was documented from the 5th to the 10th day after confrontation.

#### 2.5.2 Determination of growth inhibition percentage of FOC

Using a dual culture assay reported by Limtong et al. (2020) with slight alterations, 5 mm plugs of fungi from SDA plates were inoculated at 3 cm apart from the dish edge. Antagonistic *Bacillus* strains cultured in nutrient broth were streaked at 3 cm from the inoculated fungal plug. Control plates contained only fungal inoculum. After incubating the plates at 28 ± 2°C for 7 days, the percentage of inhibition of fungal growth by *Bacillus* strain was calculated using the following method of Limtong et al. (2020):


$$\begin{array}{l} Growth\;inhibition\;\%\;=\;(radius\;of\;fungal\;pathogen\;colony\\ cultured\;alone\;–\;radius\;of\;fungal\;pathogen\;colony\;culture\;with\\ bacterial\;strain)/radius\;of\;fungal\;pathogen\;colony\;cultured\\ alone\;\times 100 \end{array}$$

### 2.6 Extraction of lipopeptides

A solvent extraction technique was used for the initial isolation of lipopeptides from selected bacterial strains using ethyl acetate (Alajlani et al., 2016). Antimicrobial activity was checked by scratching and then dissolving the crude extract in methanol. Then, the antifungal activity was analyzed against selected phytopathogens by the cell diffusion method (Kaur et al., 2017).

#### 2.6.1 Determination of antimicrobial susceptibility by micro-dilution method

The micro-dilution method was used for the determination of the antimicrobial susceptibility of the bacterial strains. The 96-well microtiter plates were inoculated with SDA broth (70 µL) crude extract (20 µL) and fungal spore suspension (10 µL) making the total mixture 100 µL. After inoculation, microtiter plates were incubated for 30 minutes at 27°C, and absorbance was noted using an ELISA plate reader at 630 nm. Then the development was computed after 48 h at 27°C, 630 nm using an ELISA plate reader. The experiment was run in triplicate. The formula for computing the susceptibility is (Juboi, 2017).


(ΔC−ΔT)/ΔC×100

Where Δ*C =* Corrected absorbance after 48 h.

Δ*T* = Absorbance calculated after 30 min.

### 2.7 Characterization of crude lipopeptides by FT-IR

FT-IR is a fast and inexpensive technique to characterize and identify functional groups of lipopeptides produced by *Bacillus* species. The crude metabolite was extracted using solvent extraction and measurements were carried out in transmittance mode with a wavelength in the range of 400–4,000 cm (Biniarz et al., 2017).

### 2.8 Gas chromatography–mass spectrometry

GC-MS analysis was performed on the extracted metabolite to confirm the presence of both free and esterified fatty acids. The process involved hydrolytic cleavage of the links between the carbohydrate and peptide portion of the lipopeptide and the lipid portion, followed by derivatization of the resulting fatty acid chains into fatty acid methyl esters (FAME) to aid in GC-MS analysis. The samples were dissolved in methanol and filtered through a 0.22 µm pore size syringe filter (Yakimov et al., 1995).

### 2.9 *In vivo* biocontrol assay of *Bacillus* strain and its crude LPs

Chickpea seeds of the “Bittal 2016” variety were acquired from the National Agriculture Research Center (NARC) in Islamabad, Pakistan. The seeds were treated by immersing them in a solution of 5% NaOCl for 4 min, followed by multiple rinses with autoclaved water to eliminate any remaining NaOCl residues. Seeds were further disinfected with 75% alcohol and dried in a biosafety cabinet on filter paper. The soil was mixed with sand at a 2:1 proportion and autoclaved at 121°C for 21 min. Respective sterilized and un-sterilized soil was inoculated with the pathogenic fungus according to the protocol of Manghwar et al. (2021) with a few minor changes. Two seeds were sown in each pot. Five mL each of selected antagonistic *Bacillus* species was cultured in nutrient broth at 37°C and the cell-free supernatant broth was used as a bioprimer at the time of sowing. The seeds without fungal and bioprimers were regarded as negative controls and with fungus only as positive controls. Each treatment consisted of three replications including controls. The pots were placed in a greenhouse and irrigated regularly to maintain soil humidity. The chlorophyll content was measured using a SPAD-502 meter, which gives readings without damaging the leaves and, during measurement, the leaf remains attached to the plant. Harvesting was done 6 weeks after sowing. The percentage of seed germination, seedling vigor index (SVI), disease incidence, and disease control were calculated as described by Limtong et al. (2020) with an additional variable, the percentage of growth increase.


Seed germination%=(number of germinated seeds/total number of seeds planted)×100

Seedling vigor index(SVI)%=(stem height+root length)×(seed germination%)

Disease incidence%=[(SVI of negative control–SVI of positive control or treatment)/SVI of negative control]×100

Disease control%=[(disease incidence of positive control–disease incidence of each treatment)/disease incidence in positive control]×100

Growth increase%=[(SVI of treatment–SVI of negative control)/SVI of treatment]×100

### 2.10 Statistical analysis

XLSTAT^®^ software was utilized for data analysis and ANOVA was performed. To compare means, Duncan’s multiple range test was applied, and statistical significance was determined at a significance level of p ≤ 0.05.

## 3 Results

### 3.1 Isolation and molecular identification of *Fusarium oxysporum* and *Bacillus* strains

**Figure 1:**
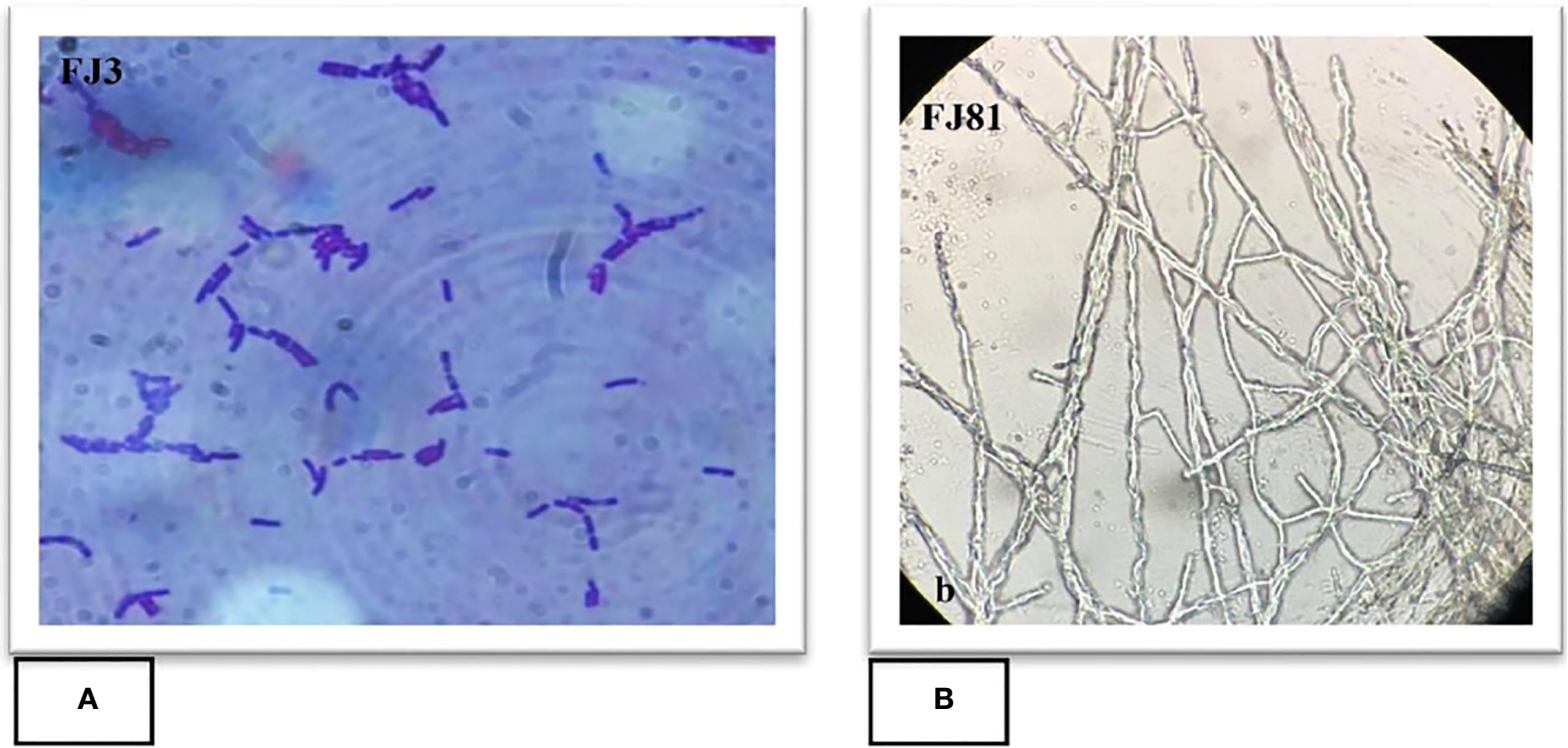
Microscopic features of **(A)** *Bacillus* strain FJ3 and **(B)** *Fusarium* strain FJ81.

**Figure 2:**
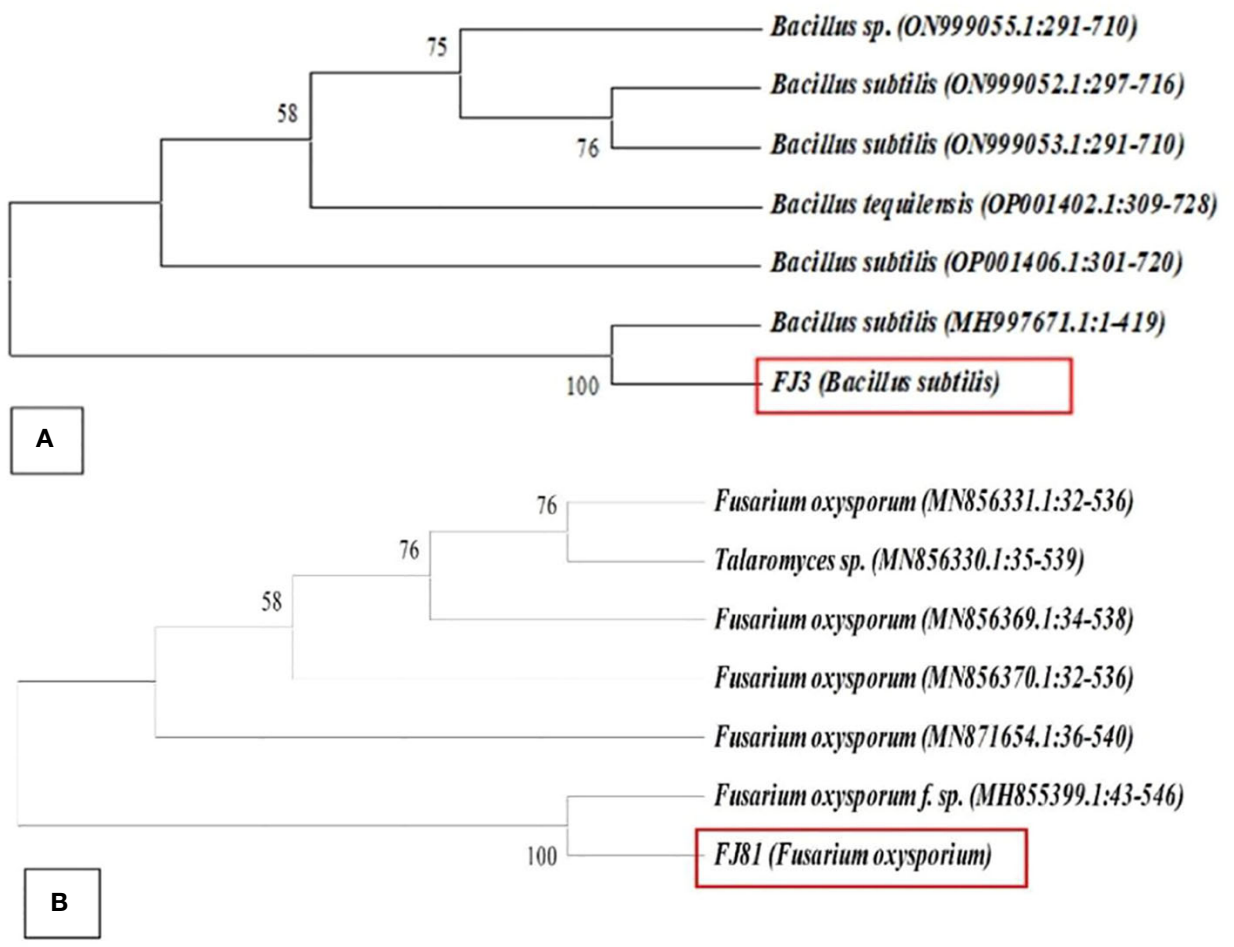
Neighborhood joining method showing the phylogenetic position of **(A)** FJ3 and **(B)** FJ81 other related taxa of the genera *Bacillus* and *Fusarium* based on 16s rRNA and 18sRNA gene sequences. Bootstrap value is > 50%.

A total of 13 purified bacterial isolates were obtained after the enrichment of the soil. These isolates were subjected to initial screening after which only one strain FJ3 was selected, as it had the greatest ability to inhibit the growth of plant pathogens. The isolated bacterial strain FJ3 and fungal strain FJ81 were further identified as *Bacillus subtilis* and *Fusarium oxysporum* via 16s rRNA and 18s rRNA gene sequencing. The taxonomic tree indicated a relationship of both *Bacillus* and FOC with later formerly identified *Bacillus* strains and *Fusarium oxysporum*; these are highlighted in colored text. The gene sequence of both FJ3 and FJ81 was submitted to the GeneBank database and accession numbers were assigned (FJ3 OQ073531 and FJ81 OQ073532). Light microscopy results of FJ3 revealed Gram-positive rods and motile and single cells. Identification characteristics are presented in **Figures 1A, B**, **2A, B**.

### 3.2 Growth-promoting traits

#### 3.2.1 Phosphate solubilization

**Figure 3:**
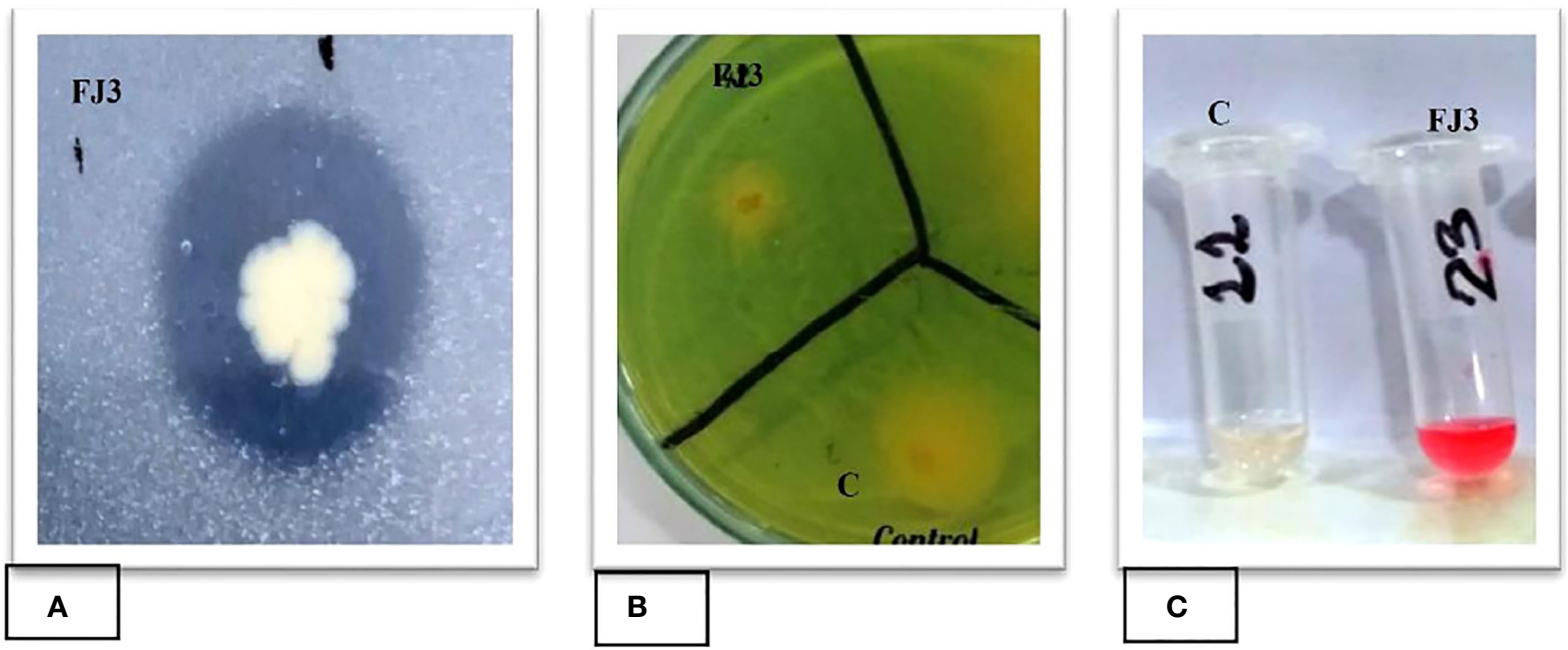
Plant growth promoting traits of strain FJ3: **(A)** Phosphate solubilization, **(B)** Siderophore production, and **(C)** IAA production.

To determine the phosphate solubilizing activity of selected bacterial strain FJ3, the strain was point inoculated at the center on NBRIP media. The results showed that strain FJ3 could solubilize tricalcium phosphate in large quantities. The halo zone around the colony indicated phosphate solubilization (**Figure 3A**).

#### 3.2.2 Siderophore production ability

Chrome Azurol Sulphonate (CAS) agar media was used to determine the siderophore production activity of both selected strains. The change in medium color from green to yellow indicates the siderophore ability of both strains (**Figure 3B**).

#### 3.2.3 Indole acetic acid production ability

*Bacillus subtilis* strain FJ3 was assessed for its indole acetic acid production ability. The FJ3 strain was inoculated in Luria Bertani broth with tryptophan. The appearance of pink color after adding Salkowski reagent in cell-free supernatant indicates a positive result. **Figure 3C** showed that strain FJ3 secreted indole acetic acid.

### 3.3 Antimicrobial traits

#### 3.3.1 Enzyme assays

**Figure 4:**
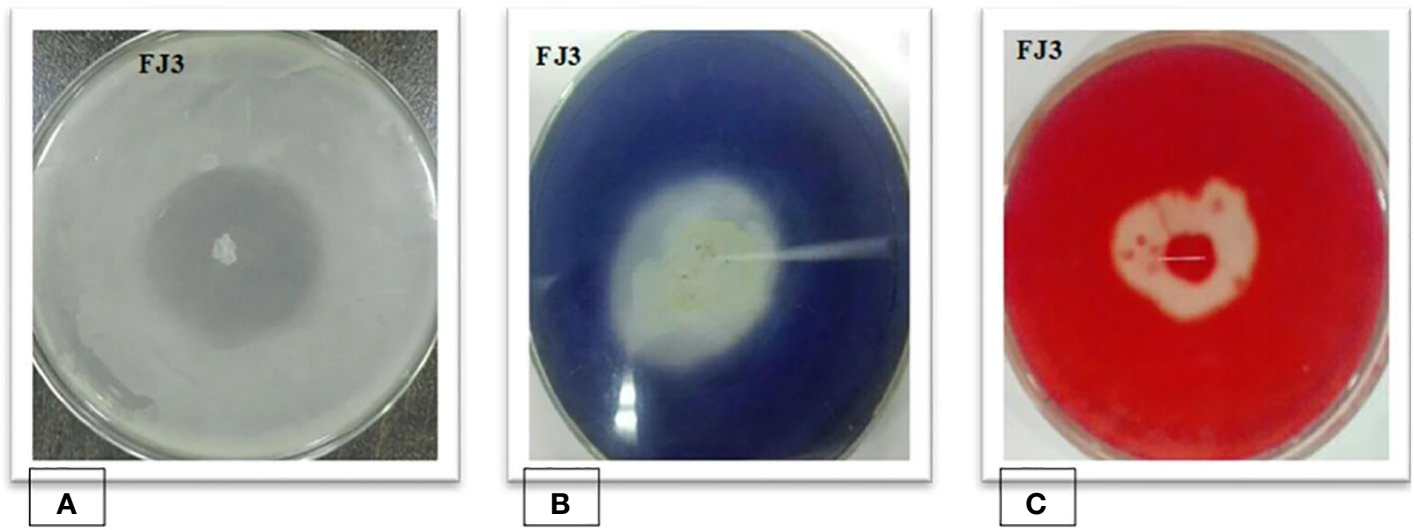
**(A)** Protease, **(B)** amylase, and **(C)** endoglucanase activity of *Bacillus* strain FJ3.

The selected *Bacillus subtilis* strain FJ3 manifested positive results for protease, amylase, and endoglucanase. Results are depicted in **Figure 4**.

#### 3.3.2 Determination of biofilm production

**Figure 5:**
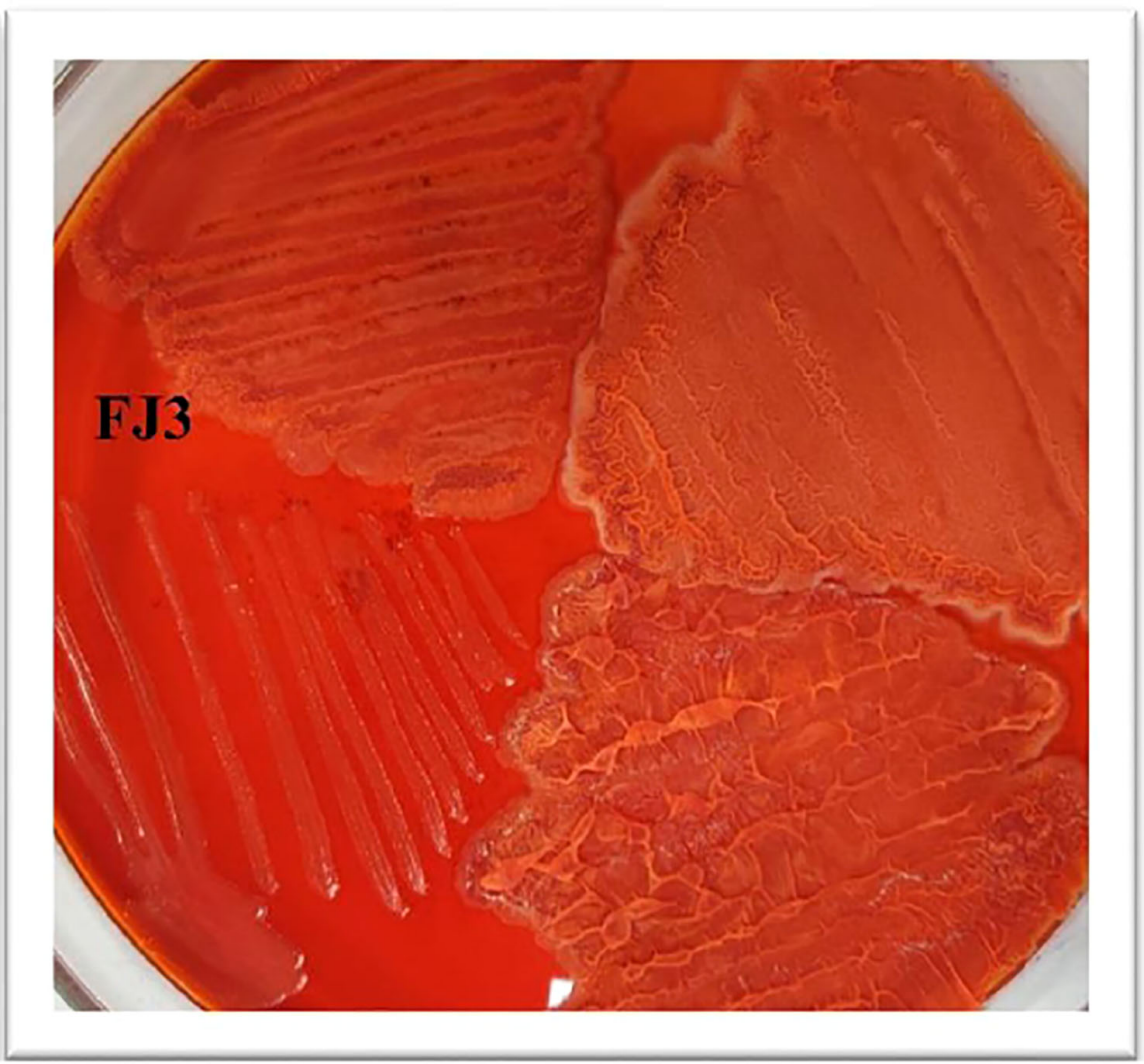
Biofilm forming ability of *Bacillus subtilis* strain FJ3.

To determine the ability of *Bacillus* strains to form a biofilm, Congo Red agar was utilized. Wild-type *Bacillus*, having the gene for TasA amyloid protein, will appear red while mutated strains will not bind to the dye. The *Bacillus subtilis* strain FJ3 appeared red, indicating its potential to produce biofilm (**Figure 5**).

#### 3.3.3 PCR analysis of NRPS gene clusters

**Figure 6:**
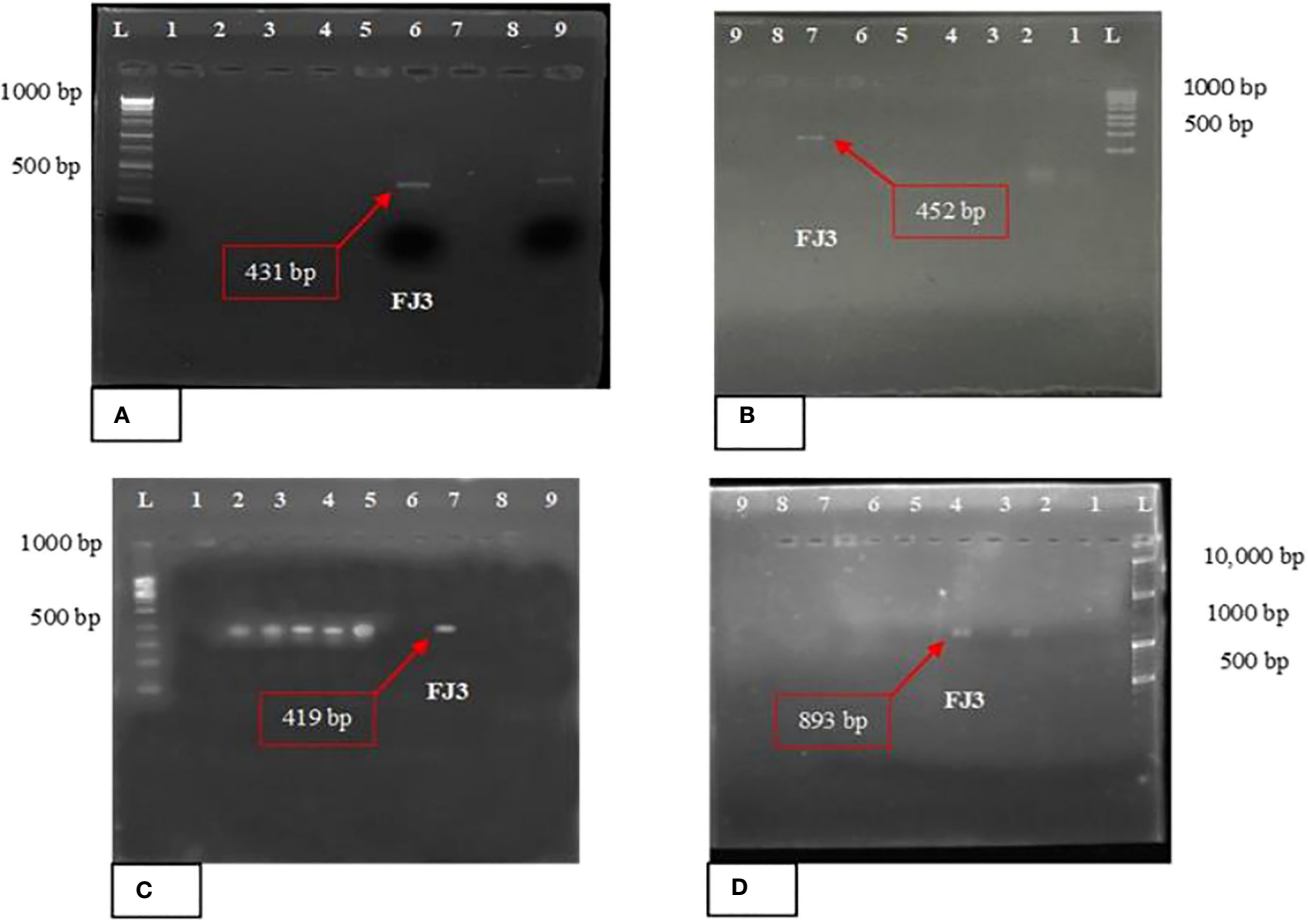
PCR amplification with different primer pairs of **(A)** surfactin, **(B)** fengycin, **(C)** mycosubtilin, and **(D)** plipastatin. *Bacillus* strain FJ3 L = Ladder of 1kb and 10kb.

Primer pairs of As1-F/Ts2-R, Af2-F/Tf1-R, Ap1-F/Tp12-R, and Am1-F/Tm1-R were used to amplify surfactin, fengycin, plipastatin, and mycosubtilin genes, respectively. The NRPS gene clusters for mycosubtilin, surfactin, and fengycin in *FJ3* were detected using multiplex PCR. In *Bacillus* strain FJ3 surfactin, mycosubtilin, and fengycin were detected in the range of 420–450 bp and for plipastatin in the range of 890–950 bp, suggesting a positive result for the presence aforesaid lipopeptides. The results of PCR testing are illustrated in **Figure 6**.

### 3.4 *In vitro* antifungal activity

#### 3.4.1 Dual culture assay against FOC

**Figure 7:**
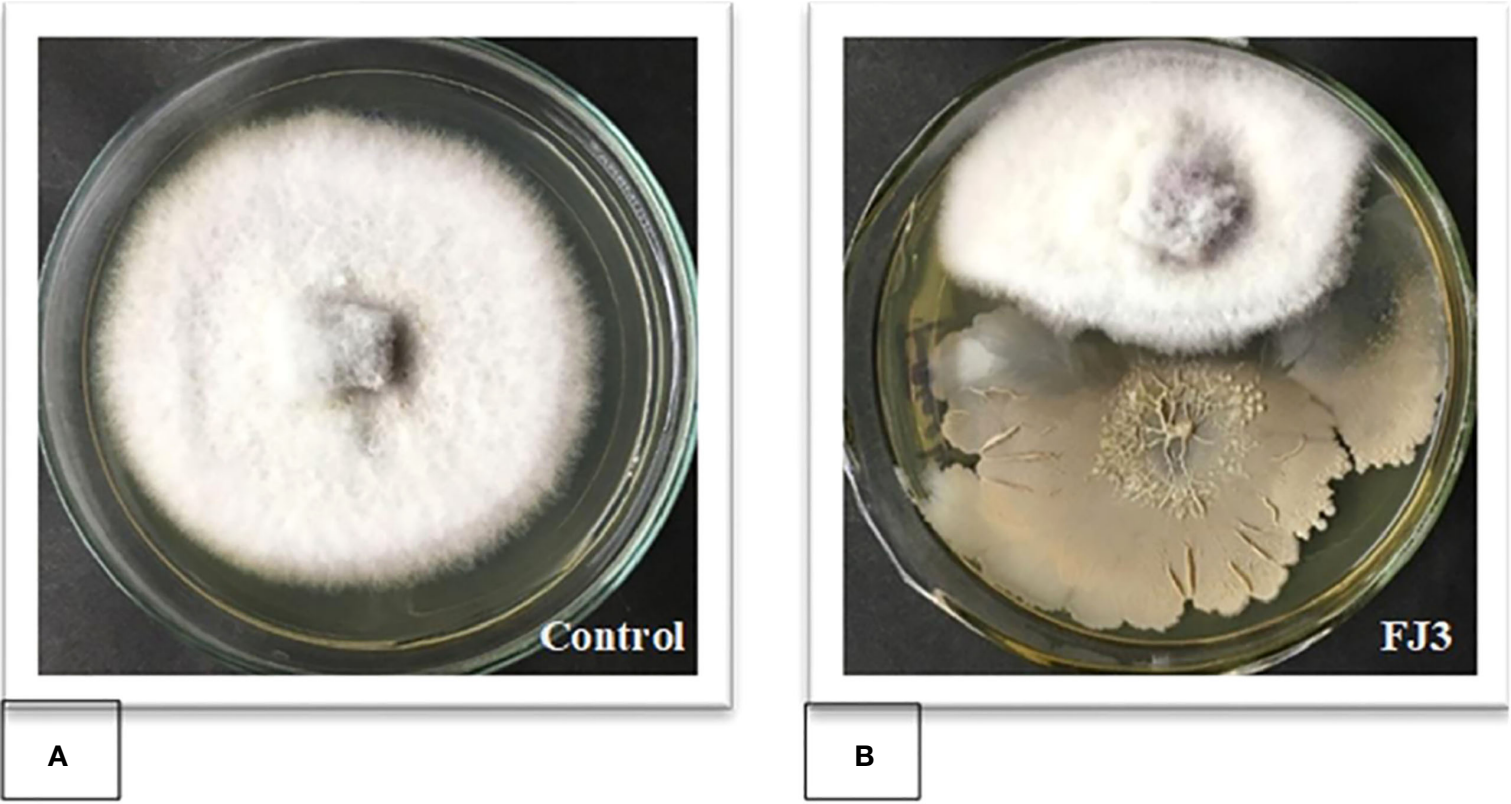
Dual culture assay of *Bacillus* strain FJ3 against *F. oxysporum* to measure the percentage of growth inhibition. **(A)** Control (*F. oxysporum*). **(B)** *Bacillus subtilis* FJ3 with *F. oxysporum*.

*Bacillus subtilis* FJ3 showed broad-spectrum antifungal activity against *F. oxysporum* and other tested fungal phytopathogens. In a dual culture assay, FJ3 inhibited 52.92± 0.72% mycelial growth of *F. oxysporum* when incubated at 28 ± 1°C (**Figure 7**).

**Figure 8:**
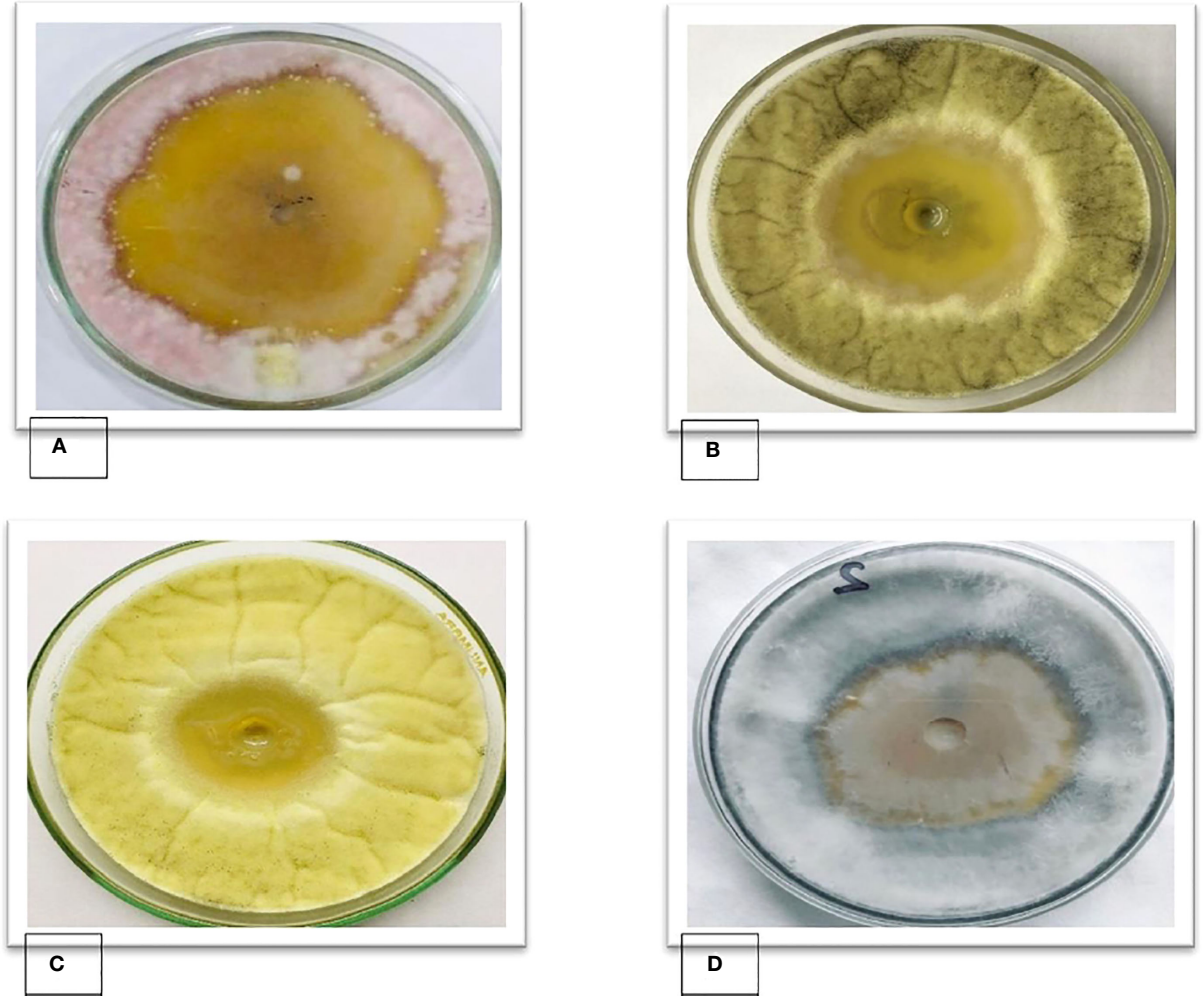
Zones of inhibition of cell-free supernatants of *Bacillus* strain FJ3 against **(A)** *F. oxysporum*, **(B)** *A. niger*, **(C)** *A. flavus* and **(D)** *R. oryzae*.

In an agar well diffusion assay *B. subtilis* FJ3 showed antifungal activity in an SDA medium against tested fungal pathogens *A. niger*, *A. flavus*, *R. oryzae*, and *F. oxysporum*. The results of *in vitro* antifungal activity indicated that the cell-free supernatant obtained from *B. subtilis* FJ3 had significantly higher inhibitory activity, 53 mm, against *F. oxysporum*, 40 *mm* for *A. niger*, 42 mm against *A. flavus*, and 38 mm against *R. oryzae* (**Figure 8**).

### 3.5 Determination of minimum inhibitory concentration

The minimum inhibitory concentration (MIC) of the crude lipopeptide extracts from strains *Bacillus subtilis* FJ3 illustrated broad anti-fungal activity with >40% inhibition against almost all tested pathogens. Growth inhibition was against *Fusarium oxysporum* (92%*), Aspergillus flavus* (81.5%), *A. niger* (90%), and *Rhizopus oryzae* (56%). The commercialized anti-fungal drug, Nystatin, showed maximum growth inhibition while no growth inhibition was manifested by methanol.

Crude metabolite FJ3 against which fungal pathogens were found susceptible (must have inhibition activity greater than 20%) was subjected to an MIC test. MIC was evaluated at a range of dilutions. The results of the calculated MIC are shown in **Table 2**. The control (nystatin) had the highest MIC value of 0.781 mg mL^−1^ against *A. flavus*, and 1.56 mg mL^−1^ against *F. oxysporum*, and *P. oryzae* and FJ3 exhibited excellent MIC against pathogenic fungal strains of *F. oxysporum* and *A. flavus* (**Table 2**).

**Table 2:** Minimum inhibitory antifungal activity of lipopeptide extract from FJ3 at various dilutions.

Fungal pathogens	MIC in mg/ml	
	Lipopeptide extract FJ3	Nystatin
***F.oxysporum***	0.781	1.56
***A.niger***	3.125	1.56
***A.flavus***	0.781	0.781
***R.oryzae***	1.56	3.125

### 3.6 FT-IR analysis of crude lipopeptide extracts

**Figure 9:**
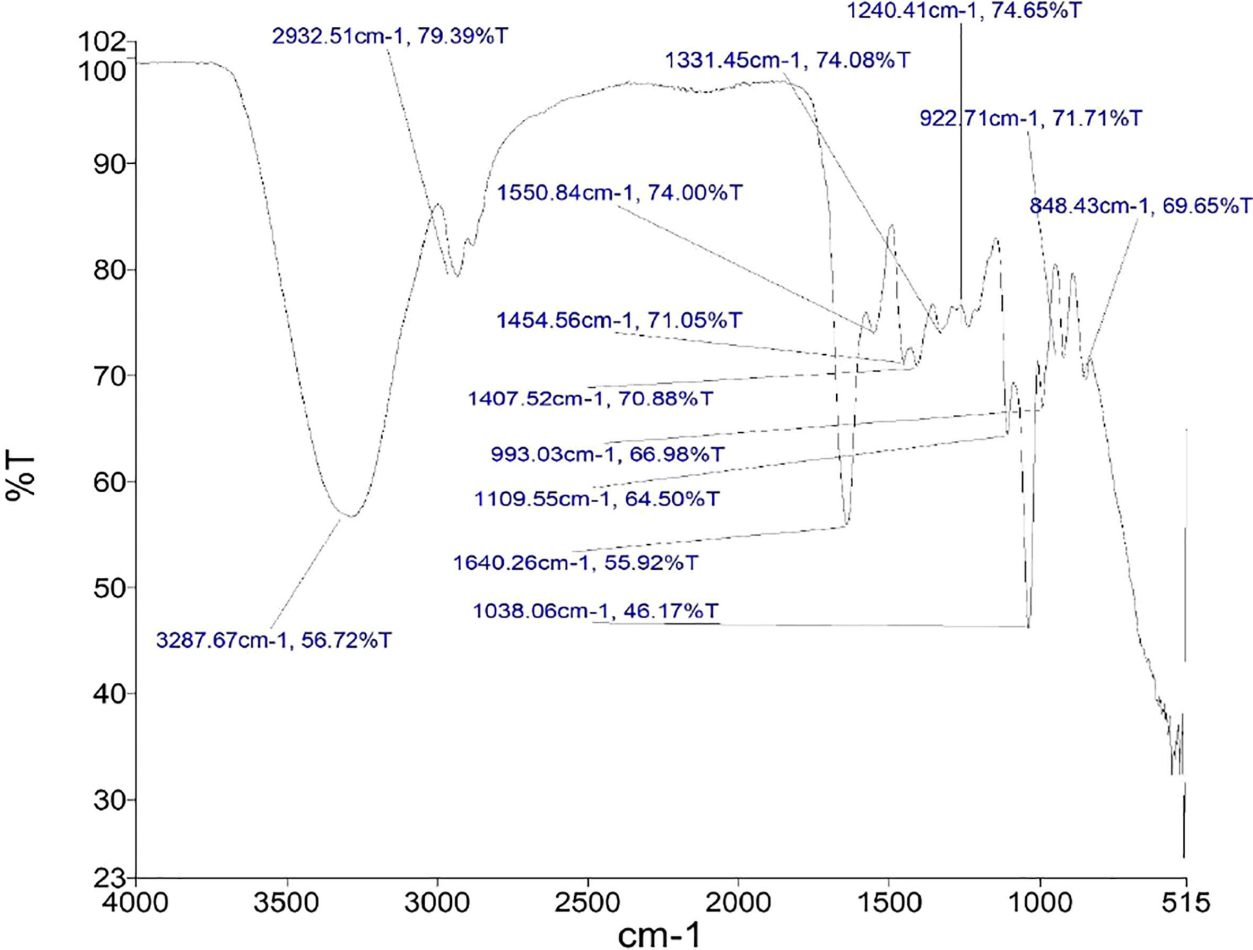
FT-IR analysis of crude extracts from *Bacillus subtilis FJ3*.

The infra-red spectrum of crude lipopeptide extracts from *FJ3* showed intense bands at 3,287.67 cm^−1^ and 3,287.94 cm^−1^, indicating the presence of an amino stretch in the metabolite architecture (**Figure 9**). Another band at 2,932.51 cm^−1^ and 2,931.49 cm^−1^ confirmed the presence of an aliphatic side chain stretch in the extract. Peaks at 1,640.26 cm^−1^ and 1,641.80 cm^−1^ corresponded to the peptide moiety while intense bands at 1,454.56 cm^−1^ and 1,455.01 cm^−1^ underlined the presence of methyl bonds and aliphatic (C-H) bonds respectively.

### 3.7 Gas chromatography–mass spectrometry

**Figure 10:**
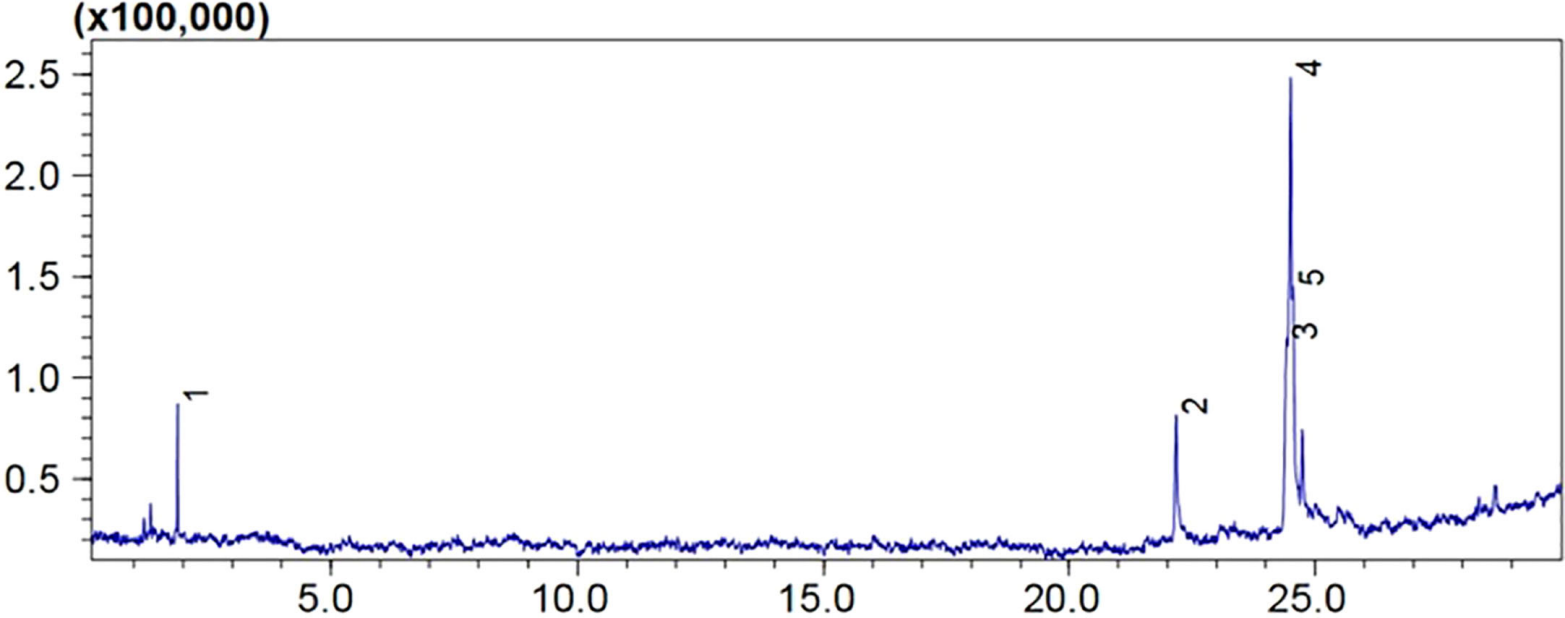
The GC-MS total ion chromatogram of fatty acids in lipopeptide isolate of FJ3. Peaks are numbered 1 to 5 as shown in the table.

**Figure 11:**
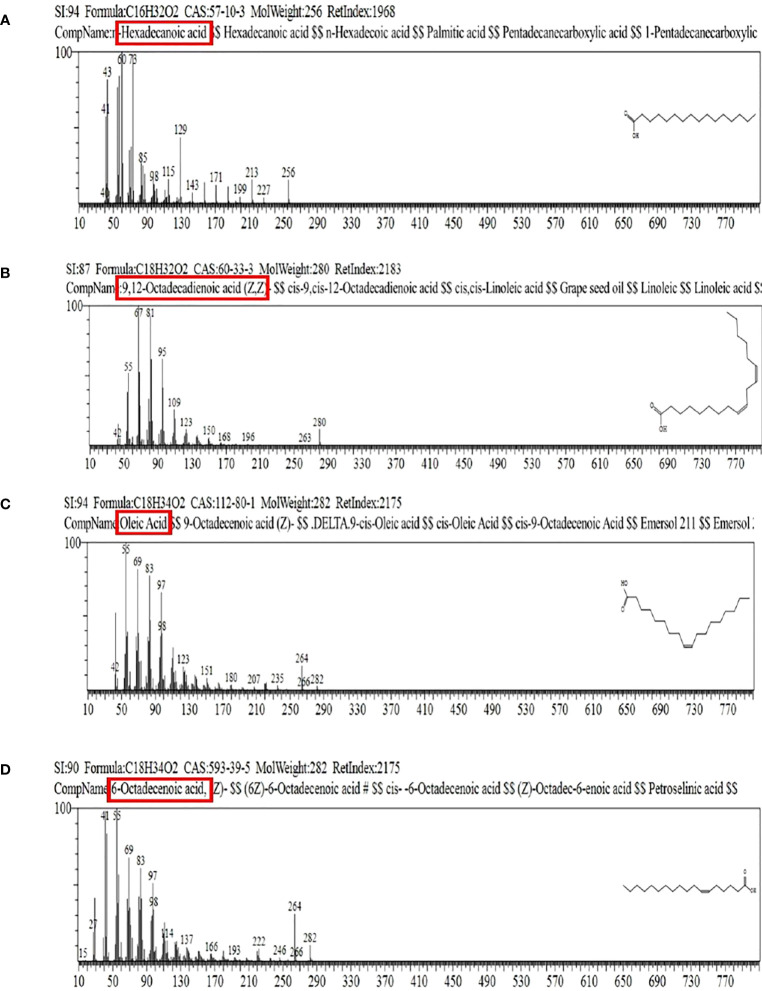
Mass spectrogram of methyl esterified fatty acid chain in lipopeptide extract of FJ3. **(A–D)** Correspond to peaks 1, 2, 3, and 4 as depicted in the figure.

Mass spectrometry analysis showed the presence of saturated β-fatty acids with chain lengths ranging from C14 to C21, and most of the fatty acids were saturated. (**Table 3**). Among the detected fatty acids, hexadecanoic acid is found to be in the majority proportion. Other detected fatty acids include oleic acid, ascorbic acids, pentadecanoic acid, 6-octadecenoic acid, and 10-heptadecenoic acid (**Figures 10**, **11**). The result is consistent with the reports of Peng et al. (2008) and Ibrahim et al. (2013).

**Table 3:** GC-MS analysis of the fatty acid composition of lipopeptide produced by *Bacillus subtilis* FJ3.

No.	Retention Time	Compounds detected	Similarity (%)	Formula
1	1.882	Trichloromethane	98	CHCl_3_
2	24.420	9,12-Octadecadienoic acid (Z,Z)-	86	C_18_H_34_O_2_
3	24.511	Oleic Acid	94	C_18_H_34_O_2_
4	24.511	6-Octadecenoic acid	93	C_18_H_34_O_2_

### 3.8 *In vivo* biocontrol assay

**Figure 12:**
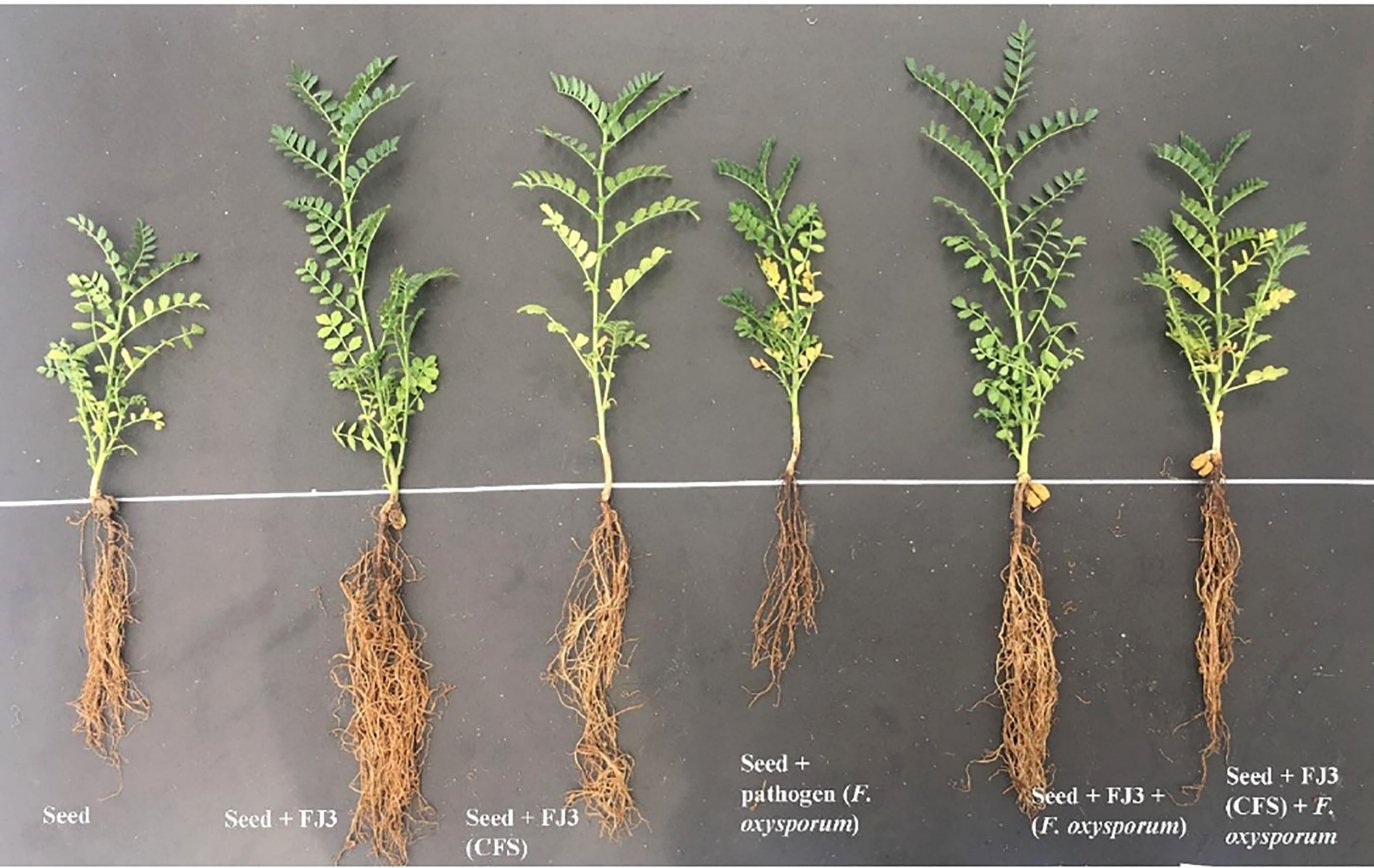
Effect of *Bacillus subtilis* FJ3 and its cell-free supernatant (CFS) treatments on Fusarium wilt of chickpea.

**Figure 13:**
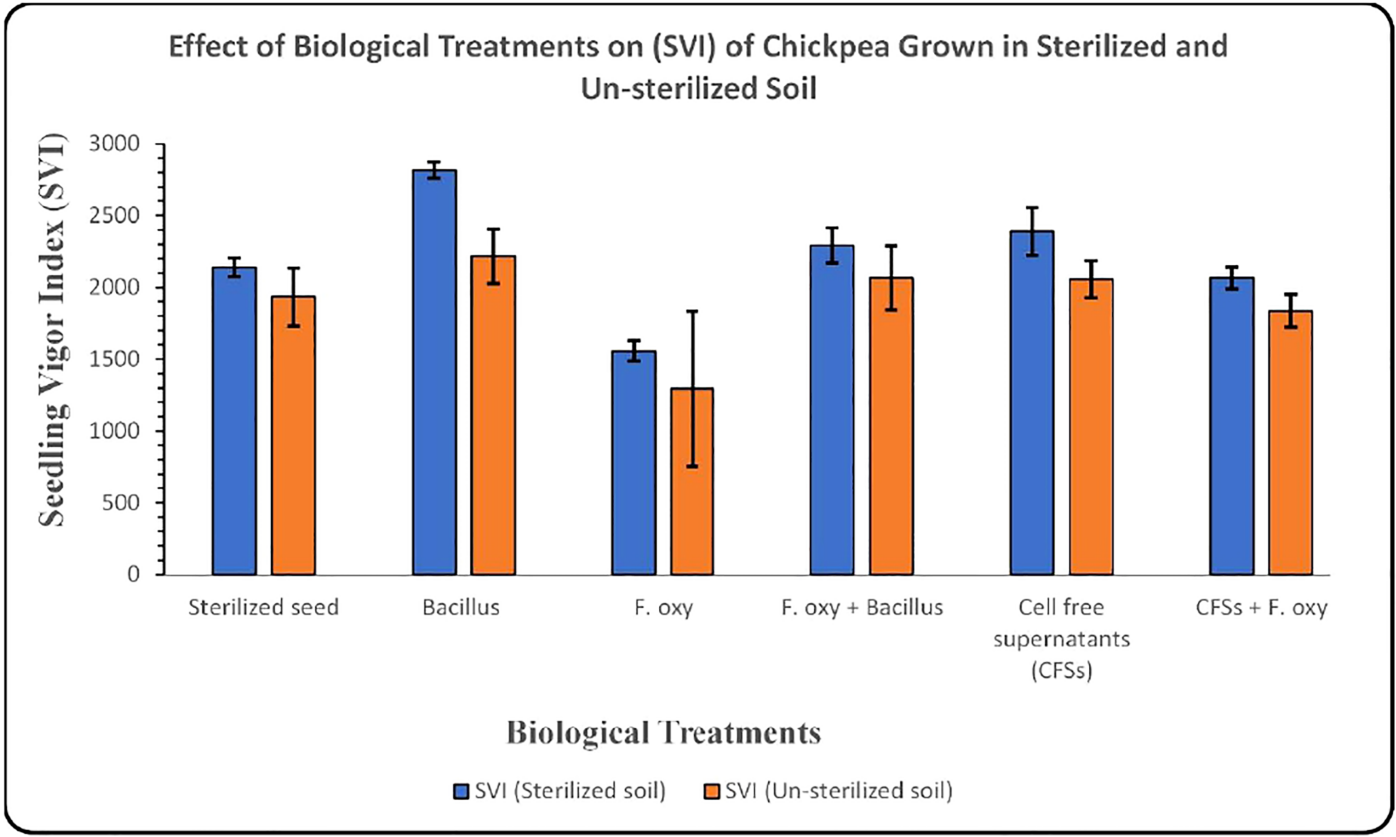
Graph showing the effect of biological treatments on the SVI of chickpea grown in sterilized and un-sterilized soil (Error bars represent standard deviations).

In sterilized soil, *Bacillus subtilis* FJ3 and its cell-free supernatant significantly controlled disease and resulted in enhanced growth as compared to the negative control. It promoted root and shoot growth appreciably with increased chlorophyll content. Although *Bacillus subtilis* and its cell-free supernatant considerably lowered the pathogen incidence in un-sterilized soil, it did not result in the promotion of growth as did the SVI for all treatments in un-sterilized soil, including controls, that were not significantly different according to Duncan multiple range test. The results are presented in **Figure 12** and **Table 4** and **Figure 13** and **Table 5**, respectively.

**Table 4:** Control of *Fusarium* wilt in chickpea caused by *Fusarium oxysporum* by *Bacillus subtilis* FJ3 and its cell-free supernatant in sterilized soil.

Treatment	Root length (cm)	Shoot length (cm)	Wet Biomass (grams)	Dry Biomass (grams)	Chlorophyll content	Germination %	Seedling vigor index (SVI)	Disease incidence %	Percent growth increase
**Sterilized seeds**	10.17 ± 0.76 bc	11.23 ± 0.75 c	2.20 ± 0.10 a	0.27 ± 0.03 a	27.20 ± 1.77 a	100 ± 0 a	2140.00 ± 65.57 cd		
***Bacillus***	12.83 ± 1.04 a	15.33 ± 1.04 a	2.81 ± 0.37 b	0.62 ± 0.46 c	39.00 ± 3.16 c	100 ± 0 a	2816.70 ± 57.74 a		24.01
***F.oxysporum***	6.63 ± 0.78 d	8.93 ± 0.81 d	1.75 ± 0.05 c	0.17 ± 0.02 d	22.67 ± 1.16 d	100 ± 0 a	1556.70 ± 70.95 e	27.26	−37.53
***Bacillus* + *F. oxy***	10.10 ± 0.53 bc	12.83 ± 0.76 b	2.23 ± 0.06 b	0.31 ± 0.02 c	30.10 ± 1.95 bc	100 ± 0 a	2293.30 ± 125.03 bc	−7.16	6.67
**Cell-free supernatants (CFSs)**	10.83 ± 0.76 b	13.07 ± 0.90 b	2.35 ± 0.05 b	0.44 ± 0.02 b	32.77 ± 1.86 b	100 ± 0 a	2390.00 ± 165.23 b		10.46
**CFSs + *F. oxy***	8.83 ± 0.76 c	11.83 ± 0.76 bc	2.07 ± 0.12 b	0.28 ± 0.02 c	29.53 ± 2.73 bc	100 ± 0 a	2066.70 ± 76.38 d	3.43	−3.58

The values presented in each column represent the mean ± standard deviation (SD). Values with different lower-case letters in the same column are considered significantly different according to Duncan’s multiple range test at a significance level of p ≤ 0.05.

**Table 5:** Control of Fusarium wilt in chickpea caused by *Fusarium oxysporum* by *Bacillus subtilis* FJ3 and its cell-free supernatant in un-sterilized soil.

Treatment	Root length (cm)	Shoot length (cm)	Wet Biomass (grams)	Dry Biomass (grams)	Chlorophyll content	Germination %	Seedling vigor index	Disease Incidence %
**Sterilized seeds**	9.50 ± 1.32 ab	9.83 ± 0.76 a	2.09 ± 0.17 b	0.26 ± 0.02 c	25.08 ± 2.76 abc	100.00 ± 0 a	1933.30 ± 202.07 a	
***Bacillus* FJ3**	11.00 ± 1.32 a	11.17 ± 0.76 a	2.79 ± 0.34 a	0.55 0.02 a	31.83 ± 3.62 a	100.00 ± 0 a	2216.70 ± 189.30 a	
***F. oxy***	7.33 ± 1.53 c	7.87 ± 0.23 b	1.70 ± 0.10 c	0.17 ± 0.02 d	20.90 ± 3.15 c	83.33 ± 28.86 a	1293.30 ± 540.49 b	33.10401903
**FJ3 + *F. oxy***	9.83 ± 0.47 ab	10.83 ± 2.02 a	2.1700 ± 0.15 b	0.28 ± 0.01 c	25.67 ± 3.06 abc	100.00 ± 0 a	2066.70 ± 223.01 a	−6.900118968
**CFSs**	9.90 ± 0.79 ab	10.67 ± 0.76 a	2.30 ± 0.10 b	0.40 ± 0.01 b	28.33 ± 4.73 ab	100.00 ± 0 a	2056.70 ± 128.97 a	
**CFSs + *F. oxy***	8.53 ± 0.42 bc	9.83 ± 0.76 a	2.07 ± 0.15 b	0.26 ± 0.01 c	23.77 ± 4.22 bc	100.00 ± 0 a	1836.7 ± 115.04 a	4.996637873

The values presented in each column represent the mean ± standard deviation (SD). Values with different lower-case letters in the same column are considered significantly different according to Duncan’s multiple range test at a significance level of p ≤ 0.05.

## 4 Discussion

The utilization of microorganisms and their metabolic products to enhance plant productivity by mitigating the effects of phytopathogens has been increasing since the beginning of this millennium. The innate ability of microbial agents to control plant pathogens, with the added advantage of enhancing agricultural productivity, has been gaining appreciation among those working in this area (He et al., 2021). The genus *Bacillus* is often more effective than other potential biocontrol microorganisms owing to its unique metabolic attributes such as the fabrication of a wide range of antimicrobial metabolites and its endospore-forming capability. *Bacillus* can accomplish the work of disease suppression through a variety of mechanisms, such as antibiosis, parasitism, competition for space and nutrients with the pathogen, or by directly inducing systemic resistance in host plants. Thus, there is a compelling demand for seeking an active bioagent that not only aids in combating disease-causing pathogens but also causes no environmental hazard (Fira et al., 2018).

The intent of the current study was to find microorganisms with highly effective anti-fungal potential and plant growth-promoting activities. This was accomplished by the isolation of soil-borne *Bacillus* strains from agricultural soils of Swat, KPK. In total, 4 out of the 13 isolates manifested good levels of anti-fungal potential against three selected test pathogens and *Fusarium oxysporum* FJ81 isolated from chickpea. Based on the zone of inhibition obtained in the preliminary spot inoculation tests, four strains FJ3, FJ9, FJ26, and FJ55 were selected as efficient strains of *Bacillus* with clear antagonism against the tested pathogens. Isolate FJ3 manifested the greatest antagonistic activity and was subjected to detailed characterization. The strain was identified as *Bacillus subtilis* by the 16S rRNA technique.

In preliminary screening, the strain *Bacillus subtilis* FJ3 displayed numerous plant growth-promoting traits such as phosphate solubilization, IAA production, siderophore production, and biofilm formation. The *Bacillus subtilis* strain FJ3 was able to solubilize phosphorus, an important feature for increasing phosphorus availability in soil for plants, to enhance crop yield. On NBRIP agar plates, the selected strain FJ3 converted insoluble tricalcium phosphate into the soluble form with a solubility index greater than 3 mm comparable with a recent study in which Javoreková et al. (2021) reported a medium solubility index value of approximately 2.96 mm for phosphate solubilization. PSBs increase phosphorus availability by secreting phosphatases and organic acids which convert phosphate to plant-available forms (Breedt et al., 2017).

The strain FJ3 was shown to produce the phytohormone IAA during an *in-vitro* assay which is involved in cell enlargement, cell division, and root growth and development, resulting in a larger root surface area allowing the plant to acquire more nutrients from the soil.

Strain FJ3 tested positive for siderophore production, indicated by the appearance of a yellowish-orange halo zone on CAS agar media (**Figure 3B**). Javoreková et al. (2021) reported that Gram-negative bacteria are primarily responsible for the formation of siderophores. Siderophores excreted by rhizosphere bacteria may promote plant growth by enhancing Fe nutrition and protecting plants against a variety of fungal and bacterial infections. Bacteria that produce siderophores can play a significant role in the biocontrol of several phytopathogens (Javorekova et al., 2020).

*Bacillus subtilis* strain FJ3 formed dry reddish colonies on Congo Red agar. Romero et al. (2010) indicated that the integrity of the extracellular matrix is strengthened by amyloid fibers formed of an amyloid protein TasA. TasA also helps in biofilm formation. TasA gives the colony a red appearance on binding with Congo Red dye. *Bacillus* species having a mutated form of the gene for TasA cannot form biofilm and colonies do not appear red. Anckaert et al. (2021) reported that biofilm formation helps *Bacillus* strains to occupy space and acquire nutrients as the mobility of nutrients is enhanced within the rhizosphere by the extracellular matrix, resulting in a reduction in the colonization of plant pathogens. According to Chen et al. (2013), restricted production of the matrix by *Bacillus subtilis* (3,610) resulted in decreased biocontrol efficacy against the *Rhizoctonia solanacearum* pathogen of tomato. Our *Bacillus* species was a biofilm producer with capacity to occupy space by inhibiting the mycelial growth of isolated fungi *in vitro* and to inhabit plant surfaces for disease prevention.

Plant growth-promoting rhizobacteria (PGPR) have been recognized for their potential to control soil-borne plant pathogens through the production of antibiotics and hydrolytic enzymes, which are recognized as key traits for biocontrol. In particular, PGPR can prevent the growth of phytopathogens by producing enzymes that can degrade fungal cell walls. A recent study has shown that strain FJ3, like other biocontrol bacteria, can synthesize enzymes such as protease, glucanase, cellulase, and pectinase. These enzymes break down specific components of the cell wall of several pathogenic fungi, such as *Rhizoctonia solani*, *Sclerotium rolfsii*, *Fusarium oxysporum*, *Phytophthora* spp., *Pythium ultimum*, and *Botrytis cinerea*. This enzymatic activity can efficiently control the spread of diseases by inhibiting the growth of these fungi (Beneduzi et al., 2012).

*Bacillus subtilis* FJ3 was also screened in specific media to produce various hydrolytic enzymes (protease, endoglucanases, and amylase). Production of these enzymes was obvious from the zone of hydrolysis during enzyme assays. Chitinases and endoglucanases destroy the basic building block of fungal cell walls, i.e., chitin and β 1,3 glucanase and β 1,4 glucanase, resulting in the loss of cell wall integrity by fungal pathogens. Production of these enzymes in *Bacillus* species was also confirmed in several recent papers (Thakaew and Niamsup, 2013; Anabella et al., 2013; Mnif and Ghribi, 2015).

PGPR primarily employ the production of antifungal metabolites and antibiotics as their key mechanisms to restrict the invasion of pathogens in host plant tissues. To investigate the antibiosis mechanisms of the FJ3 strain, PCR analysis was performed to screen four non-ribosomal peptide (NRP) genes that are responsible for synthesizing lipopeptides. Primer pairs of As1-F/Ts2-R, Af2-F/Tf1-R, Ap1-F/Tp12-R, and Am1-F/Tm1-R were able to amplify surfactin, fengycin, mycosubtilin, and plipastatin genes respectively. Fengycins and iturins exhibit potent antifungal properties and effectively inhibit the growth of various plant pathogens. Although surfactins alone do not possess antifungal properties, they have a synergistic effect with the antifungal activity of iturins (Ongena and Jacques, 2008). Results were considered positive when bands of appropriate size were obtained. The presence of surfactin genes in *Bacillus subtilis* and *Bacillus cereus* has been described by many authors; it was surprising to detect surfactin biosynthetic genes in other *Bacillus* species, i.e., *Bacillus tropicus*, *Bacillus velenzensis*, *Bacillus proteolyticus*, and *Bacillus paramycoides*. Similarly, the presence of plipastatin biosynthetic genes was observed in *Bacillus subtilis*, *Bacillus tropicus*, *Bacillus velenzensis*, and *Bacillus proteolyticus* and the fengycin biosynthetic gene were observed in *Bacillus subtilis*, *Bacillus tropicus*, *Bacillus velenzensis*, *Bacillus proteolyticus*, and *Bacillus paramycoides*. Bands of various intensities were discerned with *Bacillus* strains for each pair of primers. A band of high intensity corresponds to the amplification of many fragments of similar size. The selected *Bacillus* strain FJ3 showed positive results for surfactin, fengycin, mycosubtilin, and plipastatin genes. The amplification of expected gene size is in accordance with the work of Tapi et al. (2010). This method is highly effectual in multiplex PCR and in metagenomics.

To date, to our knowledge, this is the first report that confirms the presence of NRPS genes in strains of *Bacillus tropicus*, *Bacillus velenzensis*, *Bacillus proteolyticus*, and *Bacillus paramycoides*. *B. velezensis* is known to be a lipopeptide producer, i.e., *B. velezensis* FZB2 is known to be a PGPR (Antil et al., 2023).

The present study conducted *in vitro* inhibition assays against four fungal phytopathogens to determine the broad antagonistic ability of *B. subtilis* FJ3. The use of the *in vitro* dual culture method to evaluate potential biocontrol activities has been widely employed in previous studies. *Bacillus* strain FJ3 inhibited the mycelial growth of *Fusarium* by up to 52.92%. Mejía-Bautista et al. (2016) reported that *Bacillus* strains isolated from soil showed growth inhibition of *Fusarium solani* (3.76% to 69.16%) and *Fusarium equiseti* (2.15% to 71.55%). Using the agar well diffusion method, FJ3 showed broad-spectrum antifungal activities with maximum inhibition against *Aspergillus flavus*, *Aspergillus niger*, *Fusarium oxysporum*, and *Rhizopus oryzae*. Antifungal lipopeptides, extracted with solvents, illustrated broad anti-fungal activity with >40% inhibition against almost all tested pathogens.

Growth inhibition against *Aspergillus flavus* was 81.5%, *Fusarium oxysporum* was 92%, *Aspergillus niger* was 90%, and *Rhizopus oryzae* was 56%. A distinctive novel compound manifested antifungal activity. Previous *in vitro* studies have shown that *Bacillus subtilis* (strain RMB7) can inhibit the mycelial growth of *Fusarium oxysporum* (71%), *Rhizoctonia solani* (70%), *Pythium ultimum* (83%), and *Aspergillus flavus* (75%) (Ali et al., 2014). Similarly, another *B. subtilis* (strain BP9) was evaluated for potential biocontrol against *F. oxysporum* and was shown to inhibit mycelial growth by 62.5% (Gajbhiye et al., 2010). The high anti-fungal activity shown by FJ3 in this study was subjected to the collective effect of anti-fungal metabolites including lytic enzymes, diffusible antibiotics, and siderophores, or by high production of cyclic lipopeptides. The lipopeptides extracted showed good results with MIC values ranging from 0.78 to 3.125 mg mL^−1^ and exerted a fungicidal effect on all tested fungal pathogens. The best results were obtained against *A. flavus* and *Fusarium oxysporum* species (MIC = 0.781).

The FTIR results of crude extract evinced peaks with characteristics of cyclic lipopeptides encompassing surfactin, fengycin, and iturin. The infra-red spectrum of FJ3 crude lipopeptide content indicated intense bands at 2,853.51 cm^−1^ and 2,923.01 cm^−1^, confirming the presence of aliphatic side chain stretch in the metabolite architecture. Peaks at 1,710.18 cm^−1^ and 1,739.91 cm^−1^ indicate lactone – carbonyl absorption, while intense bands at 1,457.48 cm^-1^and 1,378.14 cm^−1^ confirmed the presence of methyl bonds and aliphatic (C-H) bonds, respectively. These bands show a chemical structure resembling lipopeptides with aliphatic chains (hydrophobic domain) and peptide moieties (hydrophilic domain). Comparison with published literature indicates the presence of surfactin (Sivapathasekaran et al., 2010; Veshareh and Nick, 2018), fengycin (Wei and Yang, 2010), and iturin (Narendrakumar et al., 2016). Based on the results of this study and the comparison of these results with the lipopeptide spectra published in the published literature, it is speculated that the compound could be lipopeptide in nature (Wu et al., 2019).

GC-MS results of isolated lipopeptides evinced various types of free and bound fatty acids. The fatty acids detected are similar to those described by Peng et al. (2008) and Ibrahim et al. (2013). Representative lipopeptides, i.e., surfactin, fengycin, and iturin, are characterized by the presence of β-hydroxy fatty acids which are crucial for the surface activity of biosurfactants. The composition of fatty acids integrated into lipopeptides relies on medium composition. Thus, the presence of the predominant hexadecanoic acid C_16_H_32_O_2_ at maximum concentration indicates the importance of this substrate for bacterial growth and proliferation (Guo et al., 2012). Nonetheless, owing to the vast structural diversity of the lipopeptides produced by *Bacillus* species, the determination of the exact fatty acid structure in cyclic lipopeptides is not well understood yet.

Although *in vitro* assays are significant in determining the antifungal properties of *B. subtilis*, it is equally vital to assess its biocontrol efficacy *in vivo*. *In vivo* experiments carried out on chickpea crops revealed that the FJ3 strain is highly effective in suppressing root rot caused by *Fusarium* spp. In autoclaved soil, our isolated *Bacillus* strain FJ3 and its metabolites (cell-free supernatant) significantly lowered the disease incidence and resulted in 24% and 10.46% increases in plant growth compared with controls, respectively. However, seed germination did not vary meaningfully. Souad et al. (2013) also demonstrated that *Bacillus* species significantly control *Fusarium* wilt in chickpea and enhanced plant growth in sterilized soil. They further noticed that seed germination was better when inoculated with only *Fusarium oxysporum* f. sp. ciceris. Posada et al. (2016) stated that cell-free supernatants either derived from endospore or vegetative cells substantially enhanced dry weight and increased shoot length of Musa plants as compared to the control. The higher plant growth promotion in the case of *Bacillus* treated seeds, as compared to metabolites, indicates possible production of plant growth-promoting substances or helping the plants in nutrient acquisition by establishing an association via biofilm production during seedling establishment. Moreover, metabolite lability (sensitive to temperature, strong acids, and bases) limits CFSs as biofertilizers used in soil (Pellegrini et al., 2020). The reduced plant growth in *Fusarium*-infested soil might be due to stress induction in the seedlings as indicated by the significantly reduced biomass and chlorophyll contents. Shoaib et al. (2018) also reported that onion infected with the soil-borne fungus *Fusarium oxysporum* f. sp. cepa showed significantly decreased (30–70%) plant growth, biomass, and chlorophyll content. Das and Roychoudhury (2014) stated that developmental and physiological variables are affected by the overproduction of ROS, leading to reduced pigments and root growth. Our study is in agreement with Baghbani et al. (2019), who noted reduced chlorophyll concentrations in maize infected with *Fusarium verticilloides*. In non-autoclaved soil, our *Bacillus* species and its CFSs significantly lowered the disease incidence by up to 39% and 29%, respectively, however, SVI, a parameter of plant growth, was not enhanced when compared with the negative control. Martínez-Viveros et al. (2010) stated that to produce a significant change in plant growth, a critical population density of beneficial microbes is needed, however, in un-sterilized soil, there is a rapid decline in bacterial population compared with sterilized soil due to competition with indigenous microbiota and predation by nematodes and protozoa. Further rapid re-colonization of bacterial communities was also observed by Marschner and Rumberger (2004) in sterilized soil. These studies justify the observed differences in plant growth in sterilized and non-sterilized soils. To concisely summarize the results of this research project, *Bacillus* species are one of the most interesting and promising options for use as biocontrol agents, demonstrating wide inhibitions against plant pathogens. Moreover, it is desirable to consider bio-controls as a green alternative to chemical pesticides to be used in formulations of commercial biocontrol products. In this way, this novel concept of bio-controls will eventually have a space outside the laboratory, and we will be able to see its fruits in present and future crop production systems.

## 5 Conclusions

The presented research has revealed that *B. subtilis* FJ3 exhibits multiple plant growth-promoting traits, including phosphate solubilization, IAA production, siderophore synthesis, biofilm formation, hydrolytic enzyme production, and lipopeptide synthesis, indicating its potential as a microbial inoculant. *In vitro* and *in vivo* investigations have also indicated that *B. subtilis* FJ3 could be a promising biocontrol agent for managing *Fusarium* wilt caused by *Fusarium* species in chickpea plants. Therefore, future studies should focus on evaluating the application of FJ3 in field trials for chickpea and other crops to facilitate the development of a commercialized product.

## Data availability statement

The original contributions presented in the study are included in the article/supplementary material. Further inquiries can be directed to the corresponding authors.

## Author contributions

FJ established the experiments, collected data, and wrote the manuscript. HA and MA were involved in the experimental setup and data collection. AJ provided intellectual guidance and reviewed the manuscript. DS provided funding, intellectual guidance, editorial input, and review of the manuscript. All authors contributed to the article and approved the submitted version.
